# Inhibitory Effects of Enoxolone on Porcine Epidemic Diarrhea Virus In Vitro and In Vivo

**DOI:** 10.1155/tbed/7319780

**Published:** 2026-08-28

**Authors:** Jie Li, Feixiang Zhu, Han Xu, Hongyue Li, Jiang Jin, Muyu Zhu, Junjie Liu, Jun Wang, Yingchun Zhou, Zongyi Bo, Haixiao Shen, Junfang Yan, Yan Cheng, Xinyue Chang, Xuelan Liu, Xianrui Zheng, Wei Li, Zongjun Yin, Liang Li, Pei Sun

**Affiliations:** ^1^ College of Veterinary Medicine, Anhui Agricultural University, Hefei 230036, Anhui, China, ahau.edu.cn; ^2^ Anhui Provincial Center for Animal Disease Control and Prevention, Hefei 230091, China; ^3^ Joint International Research Laboratory of Agriculture and Agri-Product Safety, The Ministry of Education of China, Yangzhou University, Yangzhou 225009, China, yzu.edu.cn; ^4^ College of Veterinary Medicine, Nanjing Agricultural University, Nanjing 210095, China, njau.edu.cn; ^5^ Key Laboratory of Applied Technology on Green-Eco-Healthy Animal Husbandry of Zhejiang Province, Zhejiang Provincial Engineering Laboratory for Animal Health Inspection and Internet Technology, College of Animal Science and Technology, College of Veterinary Medicine, Zhejiang A&F University, Lin’an 311300, Zhejiang, China, zafu.edu.cn; ^6^ College of Animal Science and Technology, Anhui Agricultural University, Hefei 230036, China, ahau.edu.cn

**Keywords:** autophagy-lysosome pathway, enoxolone, NF-κB pathway, PEDV

## Abstract

Porcine epidemic diarrhea (PED) is characterized as an acute and highly contagious intestinal ailment induced by the PED virus (PEDV), presenting clinical symptoms such as emesis, diarrhea, and dehydration. The occurrence of PED has imposed a substantial economic burden on the global swine farming industry. The PEDV strain exhibits a proclivity for mutations, and the immune response elicited by traditional vaccines may prove ineffective against the virus. Meanwhile, given concerns about the tolerance to existing drugs. Consequently, there is an urgent necessity to develop novel anti PEDV drugs. As a natural compound, studies have demonstrated that enoxolone possesses multiple pharmacological attributes, encompassing antitumor, antiulcer, antiviral, and anti‐inflammatory activities both in vivo and in vitro. In this study, we investigated the antiviral efficacy of enoxolone against PEDV. Enoxolone inhibits the proliferation of PEDV through multiple pathways. Initially, it degrades the nucleocapsid (N) protein via the lysosomal‐autophagy pathway. Additionally, in vitro experiments have demonstrated that Enoxolone suppresses the NF‐κB signaling pathway activated by PEDV, indicating its anti‐inflammatory properties and its ability to inhibit the inflammatory response triggered by PEDV in vitro. Furthermore, in vivo results show that enoxolone effectively alleviates the symptoms and intestinal damage caused by PEDV in piglets. Briefly speaking, our research verifies that enoxolone can inhibit the replication of PEDV both in vivo and in vitro, providing a novel direction for the development of antiviral drugs against PED.

## 1. Introduction

Porcine epidemic diarrhea (PED) is an acute and highly contagious intestinal disease in pigs caused by PED virus (PEDV) [1]. Its basic characteristics include causing watery diarrhea, vomiting, dehydration, and anorexia in newborn piglets [2]. The pathological changes of PEDV infection mainly include thinning of the intestinal wall and atrophy or even the disappearance of intestinal villi. Pigs of different strains and age groups are susceptible to infection, and clinical symptoms vary depending on the age of onset. In 2010, multiple provinces in China experienced outbreaks of highly pathogenic G2b subtype variants. There were significant antigenic differences between the variants and the G1a subtype vaccine strains, but there was a lack of effective vaccines targeting new variants [3, 4]. The persistent mutations in the PEDV genome [5] and the low efficacy in inducing mucosal immunity pose significant challenges to the prevention and control of PEDV. Studies have shown that PEDV infection induces endoplasmic reticulum stress and activates the NF‐κB signaling pathway, followed by upregulation of interleukin‐8 expression. It increases the expression of the proinflammatory cytokines IL‐1β, IL‐8, and IL‐6 [6]. This triggers intestinal inflammation and tissue damage, leading to severe diarrhea and high mortality in piglets.

The PEDV genome encodes four structural proteins (S, E, M, and N), 16 nonstructural proteins (Nsp1–16), and one accessory protein (ORF3). Through the precise collaboration of structural and nonstructural proteins, the entire life cycle, encompassing invasion, replication, assembly, and release, is completed. Among these, the S protein dominates invasion and immune recognition, while the N protein activates the p53‐DREAM pathway, leading to S‐phase arrest, promoting viral replication, and participating in viral particle assembly. The E and M proteins are involved in assembly and immune evasion, whereas various nonstructural proteins play critical roles in viral replication and innate immune antagonism [7]. Previous studies have identified compounds that exert antiviral activity by affecting viral protein stability, such as oleanolic acid degrading influenza virus hemagglutinin (HA) [8]; nattokinase degrading SARS‐CoV‐2S protein [9]; and small‐molecule compounds JNJ‐9676 and CIM‐834, which specifically target the M protein of coronaviruses and effectively inhibit the replication of SARS‐CoV‐2 and SARS‐CoV [10, 11]. The natural polyphenol compound salvianolic acid A targets the S1 subunit of PEDV’s S protein and exhibits significant antiviral activity in both in vitro and in vivo models [12]. Nevertheless, there are no commercially available chemical drugs for the treatment of PEDV infection by now; for the identified compounds, problems such as side effects and drug resistance have been recognized. There is still a necessity to explore novel compounds that can inhibit PEDV both in vitro and in vivo.

Autophagy is known as a resistance method that cells use to fight off infection. Many viruses can inhibit autophagy and thus promote their own replication. Some research has also identified autophagosome formation following PEDV infection [13]. PEDV causes full autophagy in Vero and IPEC‐J2 cells, such as increased LC3 lipidation, degradation of p62, and the formation of autophagic lysosomes. Knockdown of the lysosomal protease inhibitor chloroquine (CQ) or baflomycin A with Beclin‐1 or ATG5 blocks autophagy flux and inhibits PEDV replication [14].

Enoxolone, also known as 18β‐glycyrrhetinic acid is a product obtained by removing two molecules of glucuronic acid from the hydrolysis of glycyrrhizin, a compound in licorice, and has various pharmacological activities [15]. Research has shown that enoxolone has multiple pharmacological properties and exhibits antitumor [16], antiulcer [17], antiviral [18], anti‐inflammatory [19], and antihepatotoxic [20] activities both in vivo and in vitro. Kim et al. [21] evaluated the effect of enoxolone on the inflammatory response induced by fungal protease allergens in epithelial injury and investigated the inhibitory effect of enoxolone on mitochondrial reactive oxygen species (ROS) production in the human bronchial epithelial cell line BEAS2B. In this study, enoxolone treatment reduced cytokine production and migration of human neutrophil line HL60 by reducing mitochondrial ROS production [21]. Besides, enoxolone significantly reduced the infiltration of inflammatory cells and cytokine levels in bronchoalveolar lavage fluid (BAL) of mice treated with fungal allergens. Oral administration of enoxolone can induce the maturation of isolated lymphoid follicles in the intestinal mucosa and reduce the shedding of rotavirus [20]. More research has confirmed that enoxolone can activate the NF‐κB pathway in MA104 cells to inhibit rotavirus replication [22]. In this study, we found that enoxolone inhibits the production of proinflammatory cytokines to inhibit the NF‐κB signaling pathway and degrades the PEDV N protein through the autophagy lysosome pathway, thereby inhibiting PEDV replication in vitro. At the same time, animal experiments have shown that enoxolone can significantly alleviate clinical symptoms and intestinal damage caused by PEDV infection in vivo.

This study screened 200 compounds and found that enoxolone exhibited significant anti‐PEDV activity both in vitro and in vivo, effectively inhibiting PEDV‐induced inflammatory responses and targeting the degradation of viral N protein. These findings provide important evidence for the potential application of enoxolone in combating PEDV infection.

## 2. Results

### 2.1. Screening of Drugs With Antiviral Activity

**Figure 1:**
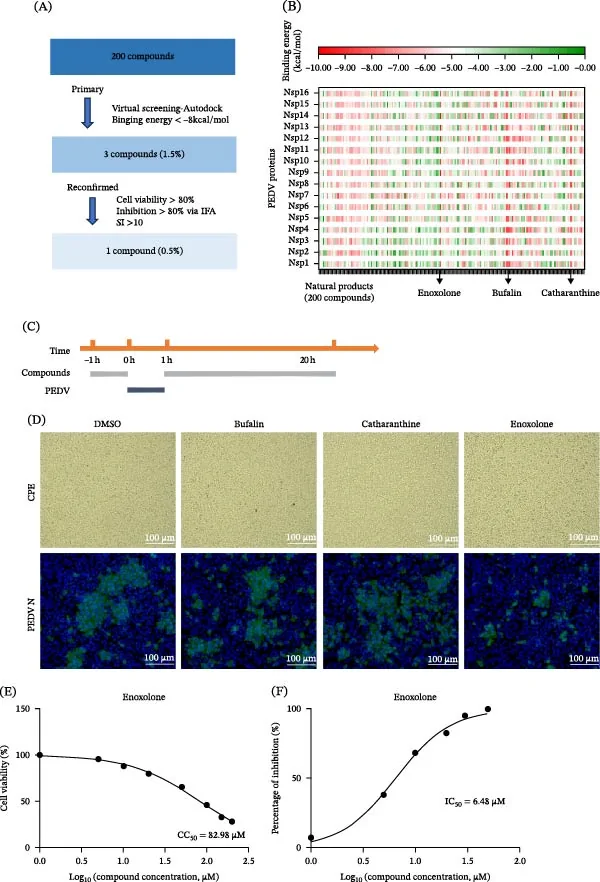
Screening of anti‐PEDV compounds. (A) The overall flowchart of drug screening. (B) Heat map for screening small‐molecule drugs. Virus proteins act as receptors, drug small molecules act as ligands, and computer software performs molecular docking to obtain the binding energy between receptors and ligands. The lower the binding energy, the more stable the binding force. Perform the next screening based on binding energy less than −8 kcal/mol. (C) Cell assay flowchart. (D) Indirect immunofluorescence detection of PEDV N protein fluorescence rate determines the final antiviral small‐molecule drug as enoxolone, and white light displays CPE. (E) CC_50_ of the enoxolone on Vero cells. (F) IC_50_ of the enoxolone on Vero cells. The CC_50_ and IC_50_ of enoxolone were compared with the DMSO group, and the effect of different concentrations of enoxolone on Vero cell activity was detected to obtain the SI (CC_50_/IC_50_) value.

Natural products are all screened using 10 μM treated Vero cells and then infected with PEDV (Figure 1A). Obtaining target drugs through three rounds of screening enoxolone. In the first round of screening, three small‐molecule drugs were preliminarily screened through molecular docking: bufalin, catharanthine, and enoxolone. The docking binding energy between enoxolone and nonstructural proteins of PEDV is mostly lower than −8 Kcal/mol (Figure 1B). The second round of screening used an indirect immunofluorescence assay (IFA) to detect virus fluorescence for these three small‐molecule drugs and found that enoxolone has the highest fluorescence inhibition rate on PEDV (Figure 1C,D). In the third round of screening, using the Cell Counting Kit‐8 (CCK‐8) method to detect the activity of enoxolone on Vero cells, the 50% inhibitory concentration (IC_50_) and the ratio of the 50% cytotoxic concentration (CC_50_) of the drug after action on the cells were measured. The selectivity index (SI) of the drug was calculated based on the ratio of the CC_50_ to the IC_50_, and statistical analysis was conducted on the results. Enoxolone exhibits a CC_50_ of 82.98 μM and an IC_50_ of 6.48 μM, SI (CC_50_/IC_50_) = 12.8 (SI > 10) (Figure 1E,F), which indicates that the safety of enoxolone is relatively high.

### 2.2. The Effect of Enoxolone on Virus Proliferation in Vero Cells and IPEC‐J2 Cells

**Figure 2:**
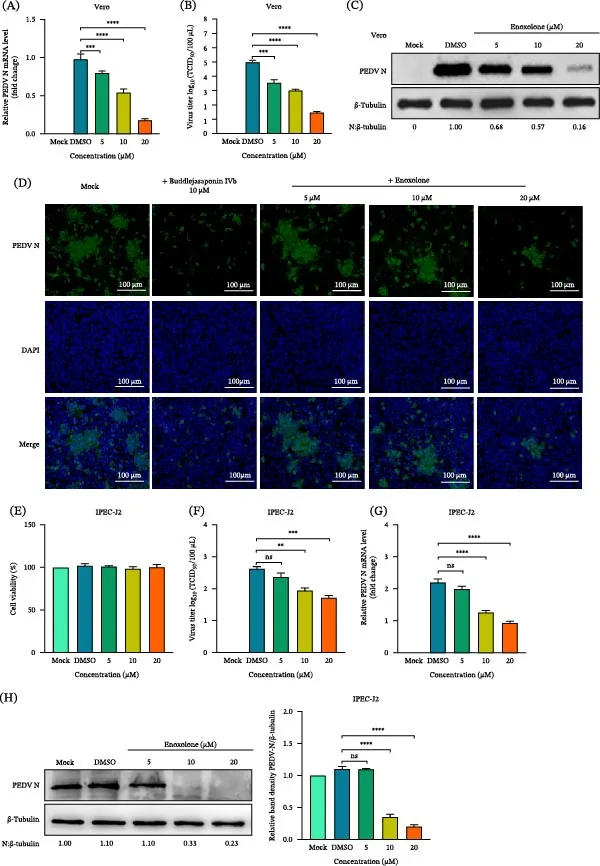
Anti‐PEDV activity of enoxolone was characterized on Vero and IPEC‐J2 cells. (A–D) Pretreat Vero with different concentrations of enoxolone for 1 h; afterwards, the cells were infected with PEDV for 1 h, washed three times with PBS, and cultured with fresh DMEM in a 37°C incubator for 20 h. The DMSO group was used as the experimental control group. (A) qPCR was used to detect the mRNA expression level of PEDV N protein. (B) Collect the supernatant from the test well and determine the TCID_50_ of the virus. Titers from three independent experiments are shown as mean ± SD (error bars). (C) After treatment with DMSO or enoxolone for 20 h, western blot was used to detect the expression level of PEDV N protein and ImageJ was used to analyze the protein grayscale level. (D) After 20 h of treatment with DMSO or enoxolone, the PEDV N protein in Vero cells was detected as green by IFA. Pretreat IPEC‐J2 cells with different concentrations of enoxolone (E–H) for 1 h; afterwards, the cells were infected with PEDV for 1 h, washed three times with PBS, and cultured with fresh DMEM in a 37°C incubator for 20 h. The DMSO group was used as the experimental control group. (E) Detection of cell activity in IPEC‐J2 treated with different concentrations of enoxolone. (F) Collect the supernatant from the test well and determine the TCID_50_ of the virus. (G) qPCR was used to detect the mRNA expression level of PEDV N protein. (H) After treatment with DMSO or enoxolone for 20 h, western blot was used to detect the expression level of PEDV N protein and ImageJ was used to analyze the protein grayscale level. These results are from one of three independent experiments. Error bars indicate SD. Asterisks in the figure indicate significant differences ( ^∗^ *p* < 0.05;  ^∗∗^ *p* < 0.01;  ^∗∗∗^ *p* < 0.001;  ^∗∗∗∗^ *p* < 0.0001).

To verify the inhibitory effect of enoxolone on PEDV through multiple experimental approaches, working solutions of enoxolone at concentrations of 5, 10, and 20 μM, or DMSO, were prepared. Vero cells were treated with these solutions for 1 h, followed by infection with PEDV at a multiplicity of infection (MOI) of 0.01 for 1 h. TCID50, qRT‐PCR, and western blot analysis showed a dose‐dependent decrease (Figure 2A–C); at 20 h postinfection (hpi), IFA showed that the number of infected cells in the enoxolone‐treated group was significantly lower than that in the negative control group (Figure 2D). To elucidate the antiviral activity of enoxolone in porcine cells, the CCK‐8 assay was initially employed to assess the impact of varying concentrations of enoxolone on the viability of IPEC‐J2 cells (Figure 2E). The results showed that a certain concentration of enoxolone had no effect on cell viability. In accordance with the experimental procedures described in the previous section regarding the impact of enoxolone on virus proliferation in Vero cells, the TCID_50_ was employed to ascertain the concentration of virus particles in the IPEC‐J2 cell supernatant (Figure 2F). The outcomes indicated that enoxolone notably decreased the content of virus particles. Western blot and qPCR analyses demonstrated that a dose‐dependent inhibition of the expression levels of the N protein by enoxolone (Figure 2G,H). All results indicate that enoxolone can inhibit PEDV replication in a dose‐dependent manner.

### 2.3. The Effect of Enoxolone on Virus Inactivation, Attachment, Entry, Replication, and Release

To further explore the mechanism by which enoxolone inhibits PEDV infection, the amount of PEDV N mRNA relative to β‐actin was measured by qPCR to determine the effect of enoxolone on PEDV inactivation, attachment, internalization, and replication (Figure 3A–E). The results showed that, compared with the control group, the transcription level of the virus during the replication stage was significantly downregulated with the prolongation of treatment time. Meanwhile, compared with the control group, the transcription level of the virus during the release phase was significantly downregulated with the prolongation of the treatment time. The results indicate that enoxolone primarily suppresses PEDV infection by influencing viral replication and release.

Figure 3Effects of enoxolone on different stages of PEDV infection. (A) Inactivated assay. Four groups: a. PEDV + enoxolone (10 μM), b. PEDV (0.01 MOI)+DMSO, c. Enoxolone (10 μM), and d. DMSO, were prepared and incubated at 37°C for 3 h. a was then mixed with d, and b was mixed with c, and the mixtures were added into Vero cells seeded in 24‐well plates. After incubation at 37°C for 1 h, culture supernatants were replaced with fresh DMEM and incubated for 12 h. The cells were washed with PBS, and the mRNA levels of PEDV N and β‐tubulin in the cells were measured using qRT‐PCR. (B) Virus attachment assay. (C) Virus internalization assay. (D) Virus replication assay. (E) Virus release assay. The results are from one of three independent experiments. Error bars represent the SD. The asterisks in the figures indicate significant differences ( ^∗^ *p* < 0.05;  ^∗∗^ *p* < 0.01;  ^∗∗∗^ *p* < 0.001;  ^∗∗∗∗^ *p* < 0.0001; ns, not significant).

**Figure:**
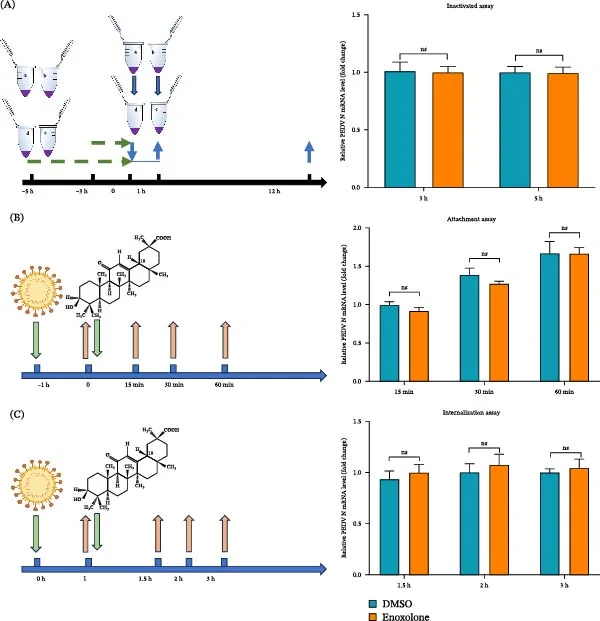

**Figure:**
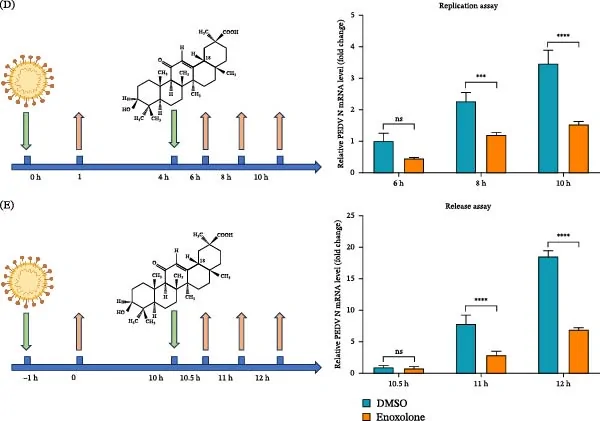

### 2.4. Enoxolone Inhibits the NF‐κB Signaling Pathway Activated by PEDV Infection

**Figure 4:**
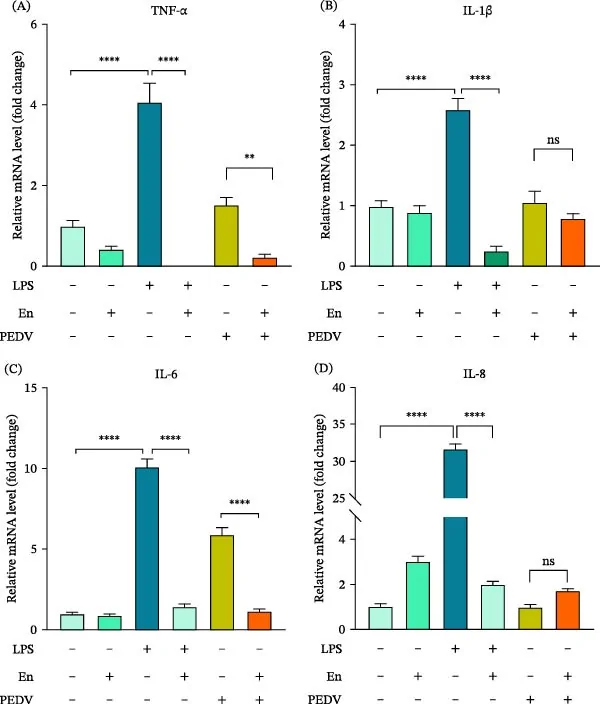
Enoxolone inhibits inflammatory factors induced by PEDV infection. (A–D) Establish a positive control group for lipopolysaccharide (LPS), which is Vero cells treated with 1 h. The control group was treated with 1 μg/mL LPS or DMEM. After coculturing the virus with cells at 37°C for 1 h, replace the virus diluent and add the preprepared working solution containing enoxolone or DMSO to the corresponding 24‐well cell plate containing cells. Continue to incubate at 37°C for 24 h to obtain the total RNA of the cells. Immediately perform reverse transcription to obtain cDNA, and use qPCR to detect intracellular proinflammatory factors TNF‐α, IL‐1β, IL‐6 and IL‐8. The transcription levels. These results are from one of three independent experiments. Error bars indicate SD. Asterisks in the figure indicate significant differences ( ^∗^ *p* < 0.05;  ^∗∗^ *p* < 0.01;  ^∗∗∗^ *p* < 0.001;  ^∗∗∗∗^ *p* < 0.0001).

**Figure 5:**
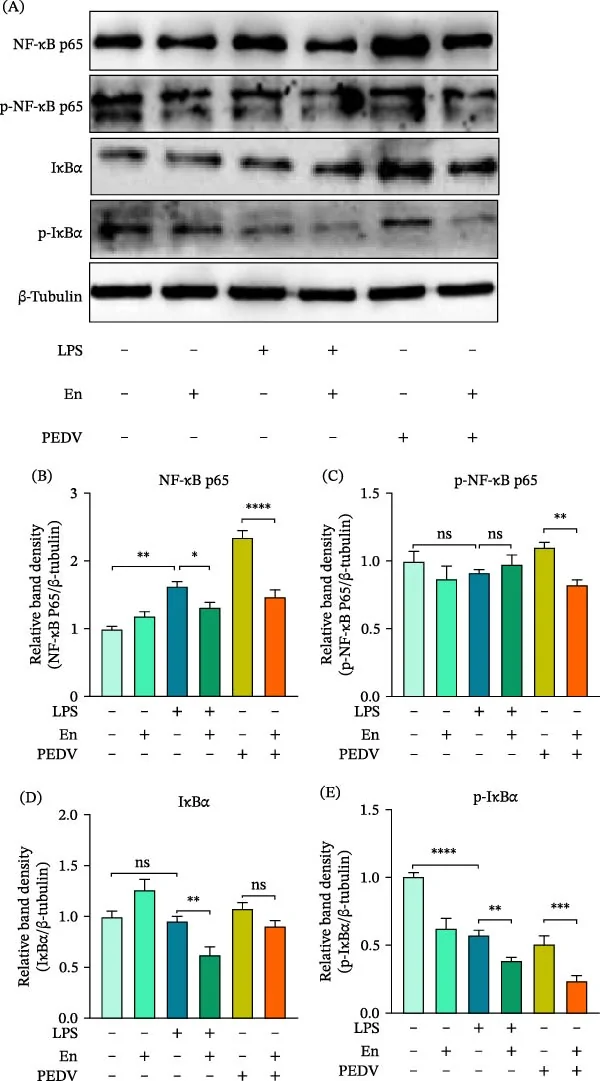
Enoxolone inhibits the NF‐κB signaling pathway activated by PEDV infection. Enoxolone inhibits the NF‐κB signaling pathway activated by PEDV infection. Set up blank control group, drug treatment group, LPS treatment group, LPS drug cotreatment group, PEDV treatment group, and PEDV drug cotreatment group. LPS stimulates cells throughout the process. In the virus attack group, the virus is first infected for a period of time, and then a working solution containing enoxolone is added to coincubate with the cells. When collecting protein samples, an appropriate amount of phosphatase inhibitors should be added to the protein lysis solution prepared in proportion. (A–E) Western blot detection of NF‐κB p65, p‐NF‐κB p65, IκBα, p‐IκBα, and tubulin, and ImageJ were used for protein grayscale analysis. These results are from one of three independent experiments. Error bars indicate SD. Asterisks in the figure indicate significant differences ( ^∗^ *p* < 0.05;  ^∗∗^ *p* < 0.01;  ^∗∗∗^ *p* < 0.001;  ^∗∗∗∗^ *p* < 0.0001).

Based on the research results above, enoxolone shows anti‐inflammatory properties, whereas PEDV infection causes severe inflammation. Set up a positive control group of Vero cells treated with 1 μg/mL LPS, while DMEM was used as the control group. The virus with cells at 37°C for 1 h, then replace the virus diluent and add the preprepared working solution containing enoxolone (or DMSO) to the corresponding 24‐well cell plate containing cells. Incubation for another 24 h at 37°C was then performed, and then the total RNA of the cells was obtanied. Immediately carry out reverse transcription to obtain cDNA, and qPCR was used to detect the mRNA level of intracellular proinflammatory factors TNF‐α, IL‐1β, IL‐6, and IL‐8 (Figure 4A–D). Results showed that enoxolone is able to reduce the amount of proinflammatory factors and virus infection in the LPS‐positive control group and virus‐infected group, respectively. To probe the character of enoxolone in the NF‐κB signaling pathway induced by PEDV infection. Western blot and ImageJ detection of intracellular p‐NF‐κB p65 and p‐IκBα after different treatments of cells (Figure 5). Cotreatment with PEDV and drugs reduced the protein expression levels of intracellular p‐NF‐κB p65 and p‐IκBα significantly. Besides, enoxolone does not increase the activation of the PEDV‐induced NF‐κB pathway.

### 2.5. Molecular Docking of Enoxolone and PEDV Proteins

**Figure 6:**
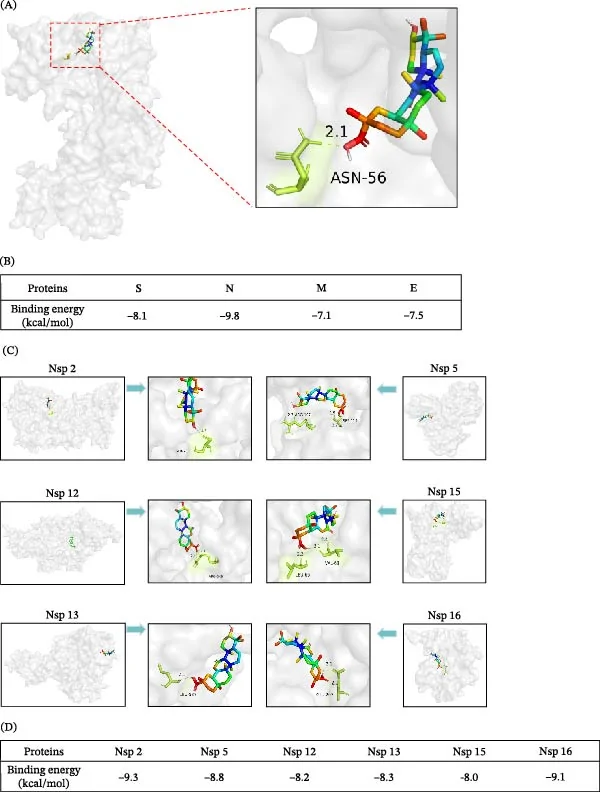
Molecular docking of enoxolone with PEDV proteins. Enoxolone exhibits high binding affinity with both key structural proteins and various nonstructural proteins of PEDV. 3D modeling of PEDV N protein using protein modeling website I‐TASSER. The binding ability of enoxolone to PEDV structural proteins was analyzed through molecular docking using Autodock Vina software. It is generally believed that the binding energy between receptors and ligands is less than −8 kcal/mol, indicating a correlation between the two. There may be a significant correlation between the binding free energy of enoxolone and the N protein of PEDV at −9.8 kcal/mol. (A) Visualization of the docking between enoxolone and N protein. (B) The docking binding energy between enoxolone and structural proteins of the virus. (C) Visualization results of the docking between enoxolone and PEDV nonstructural proteins. (D) The docking binding energy between enoxolone and nonstructural proteins of the virus.

To investigate whether enoxolone acts on PEDV directly, we used the protein modeling website I‐TASSER to perform three‐dimensional modeling of PEDV structural proteins: S protein, N protein, M protein, and E protein. The binding ability of enoxolone to PEDV structural proteins was analyzed through molecular docking using Autodock Vina software. It is generally believed that there is a correlation when the binding energy between receptors and ligands is less than −8 kcal/mol. The results are shown in the table. The binding energies of enoxolone with the structural proteins S, N, M, and E of PEDV are −8.1 kcal/mol, −9.8 kcal/mol, −7.1 kcal/mol, and −7.5 kcal/mol, respectively (Figure 6A,B). We also used Autodock Vina to analyze the binding ability between enoxolone and PEDV nonstructural proteins and visualized the interaction sites between enoxolone and PEDV nonstructural proteins using Pymol software. The results showed that the binding energy between enoxolone and multiple nonstructural proteins of PEDV was less than −8 kcal/mol (Figure 6C,D). Among them, the binding energy between enoxolone and Nsp 2 is the lowest, at −9.3 kcal/mol. The simulation results indicate that enoxolone can be a potential inhibitor affecting PEDV replication.

### 2.6. Enoxolone Degrades N Protein Through Autophagy Lysosome Pathway

**Figure 7:**
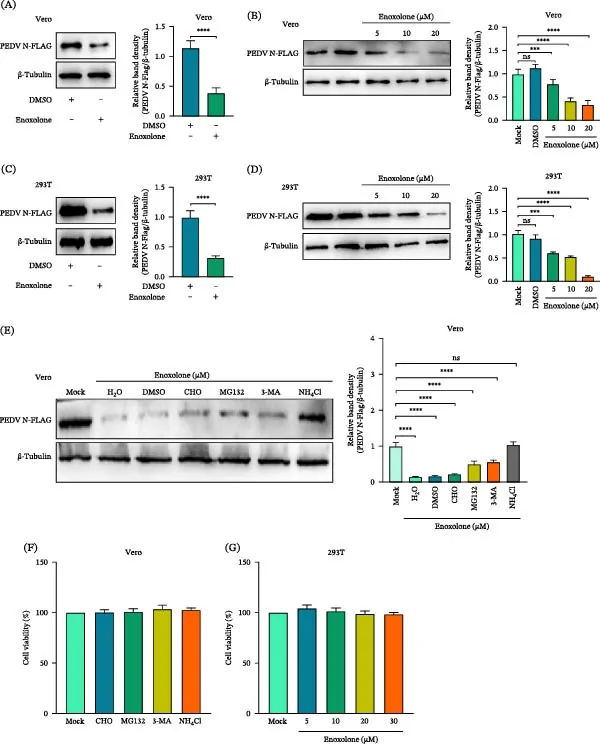
Enoxolone degrades N protein through autophagy lysosome pathway. (A, C) After transfecting pCAGGS‐flag‐N with Lipofectamine 3000 reagent into HEK‐293T cells or Vero cells for 6–8 h, change the solution to prepare DMEM with added enoxolone, mix well, and add it to the corresponding well, using DMSO as the control. Incubate in an oven at 16–18 h and then take the protein samples. After transfection of pCAGGS‐flag‐N with Lipofectamine 3000 reagent into HEK‐293T cells or Vero cells for 6–8 h, the solution was changed to prepare DMEM with added enoxolone, after thorough mixing, add to the corresponding well,with DMSO as the control. After incubation in the incubator for 16–18 h, protein samples were collected. (B, D) To further confirm the degradation of N protein by enoxolone, pCAGGS‐flag‐N was transfected into Vero cells or HEK‐293T cells in vitro for 6–8 h, the solution was then replaced with DMEM‐diluted enoxolone at concentrations of 5, 10, and 20 μM, and the DMSO group was used as the control group. After continuing to culture in the incubator for 16–18 h, protein samples were collected. (E) After transfection of pCAGGS‐flag‐N with Lipofectamine 3000 reagent into Vero cells for 6–8 h, the solution was changed to prepare DMEM with added enoxolone, after thorough mixing, add to the corresponding well. Two control groups of DMSO and H_2_O were set up. After incubation in the incubator for 14–16 h, the solution was changed to prepare DMEM with added CHO, MG132, 3‐MA, and NH_4_Cl, mixed well and added to the corresponding well. After further cultivation for 8 h, protein samples were collected. Western blot detection of PEDV N protein and tubulin content, and ImageJ were used for protein grayscale analysis. (F) The effects of CHO, MG132, 3‐MA, and NH_4_Cl on Vero cell activity. (G) The effect of enoxolone on the activity of 293T cells. These results are from one of three independent experiments. Error bars indicate SD. Asterisks in the figure indicate significant differences ( ^∗^ *p* < 0.05;  ^∗∗^ *p* < 0.01;  ^∗∗∗^ *p* < 0.001;  ^∗∗∗∗^ *p* < 0.0001).

To further delve into the way enoxolone affects the PEDV N protein, we have investigated the expression and stability of the N protein. After transfecting the pCAGGS‐N‐flag plasmid into HEK‐293T or Vero cells for 6–8 h, the maintenance medium was replaced concurrently with enoxolone at concentrations of 5 μM, 10 μM, and 20 μM, respectively; DMSO served as the control. After that, the cells were taken to 37°C for 16–18 h before collection. As shown in the western blot results (Figure 7A–D), enoxolone reduced the level of N protein in a concentration‐dependent manner by degrading it.

To explore the approximate pathway of enoxolone‐mediated N protein degradation, we selected inhibitors of different protein degradation pathways (CHO, MG132, 3‐MA, and NH_4_Cl). After transfecting pCAGGS‐N‐flag into HEK‐239T or Vero cells for 6–8 h, the medium was replaced to prepare DMEM supplemented with enoxolone and added to the corresponding wells. Two control groups were established: DMSO and water. After incubation in the incubator for 14–16 h, the medium was replaced to prepare DMEM supplemented with CHO, MG132, 3‐MA, and NH_4_Cl, which was thoroughly mixed and added to the corresponding wells. Protein samples were collected after further incubation for 8 h. Western blot results (Figure 7E) demonstrated a significant increase in the N protein content in the ammonium chloride‐supplemented group compared to the control group. Therefore, enoxolone may degrade N protein via the autophagy‐lysosomal pathway. The detection of cell viability using inhibitors at different concentrations in this study revealed no effect on cellular activity (Figure 7F,G).

### 2.7. The Therapeutic Effect of Enoxolone on Piglets Infected With PEDV Clinical Observation Results of Piglets

**Figure 8:**
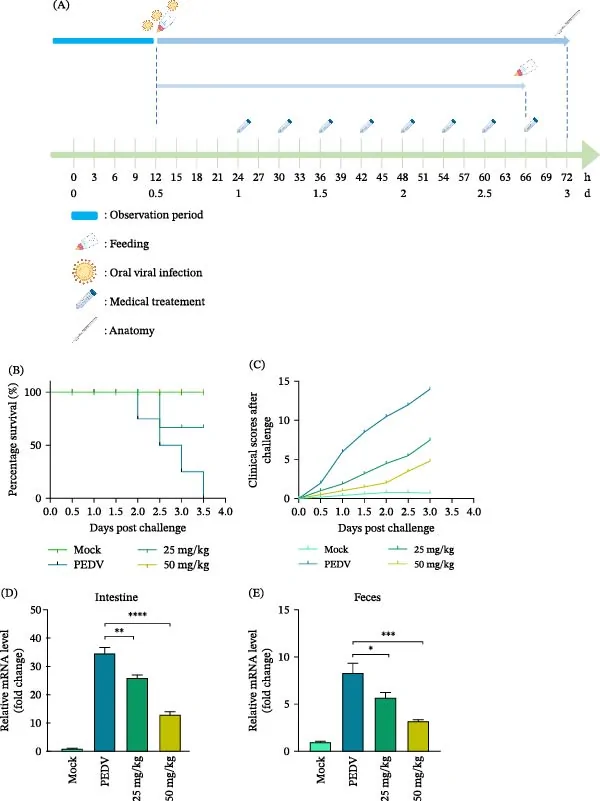
Observation of the efficacy of enoxolone on PEDV‐infected piglets. (A) Schematic illustration of the animal experimental protocol. (B) Survival rate of piglets in each group. (C) Mean clinical scores of each group were calculated. (D, E) Monitor and score the piglets daily, and analyze the viral load in porcine feces and detect enteric tissue virus content by RT‐qPCR. The first three are from the above three experiments. Error bars are standard deviations (SD). Asterisks in the graph show statistical differences ( ^∗^ *p* < 0.05;  ^∗∗^ *p* < 0.01;  ^∗∗∗^ *p* < 0.001;  ^∗∗∗∗^ *p* < 0.0001).

Regularly observe and record the clinical symptoms of piglets and count the number of piglet deaths every day. Clinical observation records: all piglets in the positive control group showed symptoms of mental depression, loss of appetite, vomiting, and watery diarrhea caused by PEDV infection. The piglets in the positive control group all died within 3 days. After 3 days of virus infection, the number of deaths and clinical symptoms of piglets were randomly observed and recorded daily (Figure 8A). The results showed that the mock group, which is the negative control group, had active and energetic piglets, increased appetite, and good growth status. The piglets in the PEDV group or positive control group all died gradually during the experimental period, and all piglets showed disheveled hair, depression, decreased appetite, and watery diarrhea. The piglets in the 50 mg/kg enoxolone treatment group showed no signs of diarrhea or vomiting and were in good mental condition. The piglets in the 25 mg/kg enoxolone treatment group experienced diarrhea symptoms on the second day after infection, and one piglet was found dead the next night. The comprehensive clinical scoring results show that compared with the positive control group, the symptoms of piglets in the drug treatment group are significantly reduced (Figure 8B,C). Therefore, enoxolone can significantly alleviate clinical symptoms caused by PEDV infection. After 72 hpi of virus infection, all surviving piglets were slaughtered, and the feces and small intestine tissues of each piglet were collected. The virus load in the tissues and feces was detected using qPCR. The viral load in the feces and intestinal tissues of infected pigs treated with the two treatment groups was significantly lower than that in the control group (Figure 8D,E). The above results indicate that enoxolone has an inhibitory effect on the replication of PEDV in pigs.

### 2.8. Pathological Changes of Piglets’ Intestinal and the Level of Inflammatory Factors in Tissues

**Figure 9:**
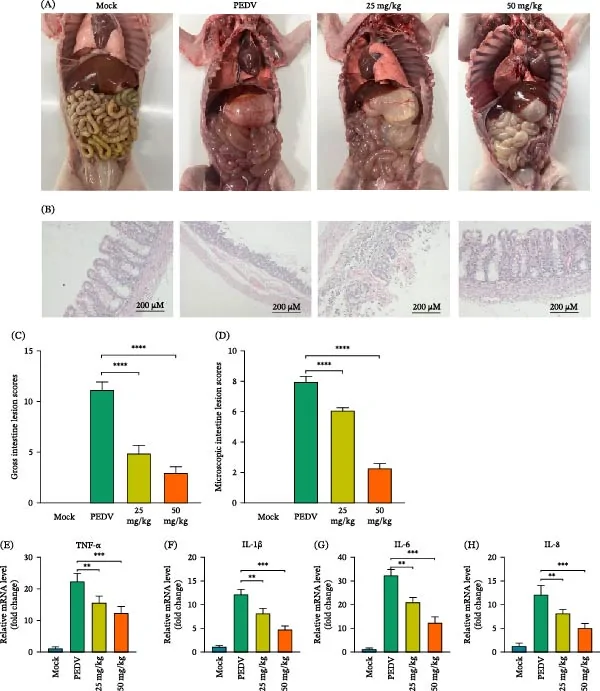
Enoxolone is capable of ameliorating intestinal lesions in piglets and decreasing the levels of tissue inflammatory factors. Establish a mock negative control group, PEDV‐positive group, 25 mg/kg enoxolone treatment group and 50 mg/kg enoxolone treatment group. (A) Pathological anatomical map of piglets. (B) Analysis of intestinal pathological sections in piglets. (C) Scores of piglet intestinal tissues. (D) Pathological section score of piglets. (E–H) qPCR detection of the expression levels of TNF‐α, IL‐1β, IL‐6, and IL‐8. The above are the results of three independent experiments. Error bars are SDs. Asterisks in the figure show a significant difference ( ^∗^ *p* < 0.05;  ^∗∗^ *p* < 0.01;  ^∗∗∗^ *p* < 0.001;  ^∗∗∗∗^ *p* < 0.0001).

After 72 hpi of virus infection, all surviving piglets were euthanized and pathologically dissected. The abdominal cavity of the piglets was opened to expose the complete intestinal tissue. The intestinal lesions of the piglets were observed with the naked eye, and scores were given based on the degree of intestinal damage. The histopathological examination of the small intestine in the attack group revealed that the small intestine was filled and dilated, the intestinal wall became thinner, and there were obvious blood streaks on the surface (Figure 9A). The intestine was filled with yellow–white liquid or milky‐white cheese‐like substance, while the intestinal tissue of the mock group piglets showed no lesions. Pathological sections of the small intestine of piglets were made, and the therapeutic effect of enoxolone was evaluated based on the thickness of the intestinal wall, the degree of shedding and damage of intestinal villi, etc.. (Figure 9B). The experimental results showed that the treatment groups with 25 and 50 mg/kg enoxolone showed significant relief of symptoms compared to the positive control group. The intestinal tissues and pathological sections of piglets were scored. Higher scores indicate poorer therapeutic outcomes in diseased piglets. The scores of the 25 and 50 mg/kg enoxolone treatment groups were significantly lower than those of the positive control group, and the scores of the 50 mg/kg enoxolone treatment group were lower than those of the drug low‐concentration treatment group (Figure 9C,D). By observing the pathological sections of piglets, results showed that the microscopic intestinal villi of the positive group pigs were significantly atrophied, and most of the long villi were broken and shed. The small intestine lesions in piglets treated with 25 and 50 mg/kg enoxolone were significantly lower than those in the positive control group under the microscope.

To determine the expression levels of inflammatory factors in intestinal tissues, RNA was extracted from the intestinal tissues of piglets in each group, and qPCR was used to detect the expression levels of related inflammatory factors in piglets infected with PEDV. The expression of TNF‐α, IL‐6, IL‐8, and IL‐1β was downregulated in the enoxolone treatment groups, which indicates that enoxolone can effectively alleviate inflammation induced by PEDV infection in the intestine of piglets (Figure 9E–H).

## 3. Discussion

Since 2010, PEDV has been prevalent in most areas of China, which has led to a high incidence rate and mortality of pigs and brought huge economic losses to the pig industry. However, the current vaccine immunization strategy is not sufficient to provide complete protection, and there is no commercialized specific drug to prevent and treat PEDV infection in pigs, indicating that the development of effective antiviral drugs is imperative.

Molecular docking serves as an auxiliary tool for evaluating receptor–ligand interactions by predicting ligand–protein binding modes based on their three‐dimensional structures. Current molecular docking techniques play a pivotal role in computer‐aided drug design. Generally, the lower the binding energy obtained, the higher the binding stability between the ligand and receptor [23]. This study utilized computer technology to establish a three‐dimensional model of PEDV proteins and small‐molecule drugs. Proteins were used as receptors, and small‐molecule drugs were used as ligands. Autodock Vina software was used to determine the free binding energy between ligands and receptors. Among 200 small‐molecule drugs, three compounds were initially screened with free binding energies of less than or equal to −8 kcal/mol with proteins of PEDV, namely, bufalin, catharanthine, and enoxolone; these three compounds were preliminarily listed as potential anti‐PEDV drugs in this study.

Subsequent experiments demonstrated that enoxolone exhibited the most significant antiviral activity and could inhibit the fluorescence intensity of the PEDV N protein. To assess the cytotoxic effects of the drug on the host or cells, CCK‐8 was used to measure and calculate the SI value. The results indicate that the CC_50_ of enoxolone is 82.98 μM, the IC_50_ is 6.48 μM, and the safety index SI value is 12.8. So, enoxolone has high safety and strong anti‐PEDV activity. To validate the reliability of simulated docking between enoxolone and PEDV N protein, pemetrexed was selected as the positive control for the simulation. The results demonstrated that the binding energy of pemetrexed to the PEDV N protein was −8.5 kcal/mol, indicating that enoxolone exhibits superior binding affinity to PEDV compared to pemetrexed (Figure S1). We did not conduct additional in vitro or in vivo experiments to corroborate this finding. It would be necessary to select previously reported compounds as positive controls to conduct deeper investigations and adjust their suitability in further studies. Our current results demonstrate that enoxolone can specifically target the PEDV N protein. Reports showed that enoxolone plays a variety of biological roles, including antiasthmatic, liver protection, anticancer, kidney protection, antidiabetes, antibacterial, antischistosomiasis, antiosteoporosis, antiepilepsy, antiarrhythmia, and anti‐inflammatory, as well as the treatment of pulmonary hypertension, antipsychotic drug‐induced hyperprolactinemia, and cerebral ischemia [24]. Hardy et al. [25] confirmed that after completing the adsorption of rotavirus, enoxolone was added to the culture for further cultivation. Finally, it was determined that the viral proteins VP2, VP6, and Nsp 2 decreased by 99%, indicating their antiretroviral activity [25]. Meanwhile, there is research that indicates glycyrrhizin acid significantly reduces SARS‐CoV‐2 replication by inhibiting the viral Mpro [26]. A randomized clinical trial on COVID‐19 patients evaluated the efficacy of nebulized glycyrrhizin–enoxolone combination, which found the therapy can effectively regulate IL‐17A levels and improve patients’ inflammatory status [27].

The life cycle of a virus includes four stages: adsorption, entry, replication, and release. Any obstacle in this process may inhibit viral infection. For example, after treating cells infected with PEDV, erastine significantly inhibited the replication stage of the virus. Further research has shown that this drug downregulates the expression of classic iron removal genes (NRF2, ACSL4, and GPX4), downregulates the iron removal pathway, and inhibits PEDV replication [28]. Also, it has been reported that salinomycin can inhibit the adsorption and entry of PEDV and significantly reduce its in vitro infection [29]. Peroxyergosterol has multiple stages in the life cycle of PEDV, including entry, replication, and release. However, it cannot inactivate PEDV particles in vitro and inhibits the infection of PEDV in Vero cells by inhibiting ROS production and p53 activation [30]. In addition, some scholars have confirmed that levonolactone A can also reduce the proliferation of PEDV. Further testing shows that coincubation with PEDV can inactivate the virus from the source [31]. Our laboratory has also done a lot of work in the research and development of anti‐PEDV drugs in the early stage and found that Buddlejasaponin IVb inhibits virus proliferation by inhibiting the replication and release stages of PEDV. Therefore, referring to the research approach of the former, this experiment was also explored through techniques such as western blot and qPCR. The effects of enoxolone on various stages of the life cycle of PEDV were studied. The results showed that enoxolone did not affect the activity of viral particles but significantly downregulated the viral transcription and protein expression levels during the replication and release stages. In response to this phenomenon, we subsequently attempted to explore the cellular pathways and related mechanisms that the drug may be involved in regulating during the virus replication and release stages.

The NF‐κB signaling pathway is a typical proinflammatory signaling pathway in which cell proinflammatory factors such as TNF‐α, IL‐1β, and IL‐6 can activate the NF‐κB signaling pathway [32]. NF‐κB is the key regulatory factor in the NF‐κB signaling pathway. In a resting state, NF‐κB, through communication with IκBα, binds to retain it in the cytoplasm, thereby inhibiting NF‐κB from entering the nucleus. The activation of NF‐κB is usually caused by the NF‐κB signaling pathway induced by activators NF‐κB suppressor protein IκBα. This is caused by rapid degradation of NF‐κB, followed by transfer of dimer p50/p65 to the nucleus to activate host gene transcription and phosphorylation of p65. Many different types of viruses can induce activation of NF‐κB signaling pathways, such as NDV, RV, and ASFV [33, 34]. In addition, the NS1 protein of porcine parvovirus (PPV) can upregulate the expression of IL‐6 and TNF‐α in a dose‐dependent manner and induce IκBα. Phosphorylation leads to phosphorylation and nuclear translocation of NF‐κB [35]. To the same, in the infection process of PEDV, the NF‐κB signaling pathway is also involved. This study confirms that PEDV infection can activate the NF‐κB pathway, while Enoxolone inhibits activation of the NF‐κB pathway. Afterwards, we measured the changes in the inflammatory pathway and cytokine levels mediated by enoxolone on PEDV‐infected cells. The qPCR results demonstrated that enoxolone significantly reduced the transcription levels of cytokines (TNF‐α, IL‐6, IL‐8, and IL‐1β) upregulated following PEDV infection. Meanwhile, western blot detected the effect of enoxolone on PEDV‐mediated key downstream proteins, NF‐κB p65, p‐NF‐κB p65, IκBα, and p‐IκBα of the NF‐κB pathway. The experimental results show that it inhibits the activation of the NF‐κB pathway induced by PEDV.

Autophagy is a highly conserved intracellular degradation pathway in eukaryotes that can break down unused cytoplasmic components into nutrients and play a crucial role in virus replication. Some studies have shown that autophagy can inhibit virus replication in human breast cancer cells to inhibit cell death induced by NDV [36]. The host protein SNX32 interacts with the viral protein CP204L and degrades CP204L by upregulating the autophagy‐related protein RAB1B, thereby antagonizing the replication of ASFV [37]. PEDV infection upregulates the RNA‐binding protein polypyrimidine bundle‐binding protein 1 (PTBP1), which targets the degradation of the virus nucleocapsid N protein through autophagy and proteasome pathways [38]. In our study, we accidentally discovered that enoxolone can degrade ectopic‐expressed N protein. To seek its potential reasons, we used the corresponding inhibitors based on the main degradation pathway of eukaryotic cell proteins. Compared with the control group, after treatment with NH_4_Cl, which mainly plays a role in the binding process between lysosomes and autophagic vesicles, significantly increased N protein. Our results indicate that enoxolone degrades PEDV N protein through the autophagy lysosome pathway, thereby inhibiting PEDV proliferation in cells. Unfortunately, we have not further elucidated its regulation of the autophagy lysosome pathway, which is worth further exploring the possible related mechanisms in the future.

Gupta et al. [39] established a chronic unmeasurable mild depression (CUMS) rat model and used oral treatment with 20 mg/kg and 50 mg/kg enoxolone doses, respectively; the results showed that the drug downregulated proinflammatory cytokines in CUMS rats and improved their depressive behavior. Caglayan et al. [40] showed that based on the rat disease model established by the toxic substance bisphenol A (BPA), oral doses of 50 mg/kg and 100 mg/kg enoxolone were selected, respectively, as well as two different concentration drug treatment groups, significantly improving symptoms related to BPA‐induced brain neurotoxicity in rats. Other scholars have administered daily administration of 50 or 100 mg/kg enoxolone to neonatal hyperoxia‐exposed rats to inhibit the activation of NF‐κB and NLRP3 inflammasomes, reduces ROS levels and pulmonary inflammation, improves alveolar development, and increases the weight of newborn rats exposed to high oxygen, confirming the protective effect of this drug on such newborn rats [41]. Based on the above research reports, we adopted oral administration and further explored enoxolone within the safe concentration range (25 and 50 mg/kg drug doses), for its therapeutic effect on piglet disease models.

On the basis of the piglet PEDV infection model established by our team in the early stage, different doses of enoxolone were orally administered for treatment, and we found that it can effectively alleviate clinical symptoms and intestinal damage caused by PEDV infection. It improved the survival rate of PEDV‐infected piglets, reduced the viral load in the ileum, jejunum, and anal swabs, and alleviated the pathological changes in the ileum and jejunum. Previous findings show the antiviral effect of enoxolone mainly comes from immunoregulation rather than the eliminating virus directly. Our study demonstrates that enoxolone inhibits PEDV proliferation by degrading PEDV N protein through the lysosomal‐autophagic pathway. Also confirms that enoxolone can inhibit the NF‐κB signaling pathway induced by PEDV, thus diminishing the inflammatory response caused by PEDV. This mechanism of degrading key viral structural protein N protein brings a new point of view and target for enoxolone’s antiviral effect, which is different from well‐known mechanisms that mainly focused on regulating host immune pathways or directly inhibiting viral replication. This study has clearly confirmed the effectiveness of enoxolone in treating PED, and its notable antiviral effect has been verified both in vitro and in vivo. Results show that enoxolone can effectively alleviate clinical symptoms and intestinal damage of piglets infected with PEDV, reduce virus content in feces and intestinal tissues, and improve survival rate. PEDV causes huge economic losses to the global pig industry, and this study provides a potential therapeutic drug for it, which has great practical application value. Unfortunately, enoxolone was not administered to healthy piglets not infected with PEDV in this study, so it cannot elucidate the preventive effect of this drug on PEDV. In the later stage, this part will be one of the research focuses to further explore the antiviral effects of enoxolone in the prevention and control of PEDV and other coronaviruses.

In summary, enoxolone, screened from a small‐molecule drug library, demonstrated effective inhibition of PEDV infection in both in vitro and in vivo experiments. This exhibits significant antiviral effects during the replication and release stages of the virus. Also, it has high binding affinity with both key structural proteins and various nonstructural proteins of PEDV. From a mechanistic perspective, this drug inhibits PEDV‐induced activation of the NF‐κB pathway and reduction of inflammatory cytokine transcription levels, utilizing the autophagy lysosome pathway to degrade the PEDV N protein, thereby inhibiting PEDV proliferation. The aforementioned research findings contribute to a deeper understanding of the pathogenesis of PEDV infection, providing natural compound candidates that directly target the N protein for PEDV prevention and control. This lays an experimental foundation for the subsequent development of antiviral drugs targeting PEDV and coronaviruses, highlighting the developmental value of enoxolone as an antiviral agent. The findings also suggest potential applications to other coronavirus infections, promote the use of natural products in the field of antiviral therapy, and provide experimental evidence for the treatment of PEDV infection in swine farming.

## 4. Materials and Methods

### 4.1. Cells, Virus, and Drugs

Vero cells, IPEC‐J2 cells, and HEK‐293T cells were preserved in Dulbecco’s modified Eagle’s medium (Gibco, USA) containing 10% fetal bovine serum (Lonsera, Uruguay). Cells were cultured in a humidified incubator (Thermo) at 37°C with 5% CO_2_. PEDV AH‐2018‐HF1 (Genebank: MN315264.1) was stored in the Animal Infectious Disease Laboratory of Anhui Agricultural University. All drugs are bought from the Selleck official website; enoxolone at > 99% purity was used for in vivo and in vitro experiments (Selleck Chemicals, USA).

### 4.2. Screening Antivirus Drugs

This study selected 200 small‐molecule drugs from the purchased FDA‐approved drug library and obtained the target study drug through molecular docking screening, enoxolone. In vitro experiments were conducted to calculate the half effective concentration (IC_50_, concentration of compounds was 1, 5, 10, 20, 50, and 100 μM) and half lethal concentration (CC_50_, concentration of compounds was 1, 5, 10, 20, 50, and 100 μM), of each remaining candidate compound were calculated using the log (inhibitor) vs. response‐variable slope (four parameters) method by GraphPad Prism 9.0 software (GraphPad Software, CA, USA), and the drug SI = CC_50_/IC_50_ was obtained to determine drug safety. Further research was conducted on natural products with a SI greater than 10 and dose‐dependent inhibition of PEDV. Besides, cell viability was assayed using the enhanced CCK‐8 (Beyotime, China) according to the manufacturer’s instructions, and the negative control was DMSO.

### 4.3. IFA

The effect of enoxolone on PEDV infection in Vero cells was assayed with IFA. DMSO‐dissolved enoxolone (concentrations of 5, 10, and 20 μM) was added to the cell culture medium. The negative control was DMSO. Then, PEDV (0.01 MOI) infected cells were incubated at 37°C for 20 h. The cells were then fixed with 4% paraformaldehyde for 20 min, washed with PBS, and then permeabilized with 0.1% Triton X‐100 at 37°C for 20 min. The treated cells were incubated with mouse anti‐PEDV N protein multiplexes prepared in our laboratory (1:100 dilution) at 37°C for 2 h. PBS was washed 3 times and added to FITC‐labeled goat anti‐mouse IgG (H + L) antibody (1:200 dilution) (A0568, Beyotime, China) at 37°C for 1 h. Cells were observed with a Zeiss inverted fluorescence microscope after PBS washing. The fluorescence‐integrated optical density of PEDV N was measured via ImageJ software.

### 4.4. Virus Inactivated Assay

Enoxolone (20 μM) or DMSO was added to PEDV (1 MOI) and then kept at 37°C for 3 and 5 h. A mixture of enoxolone or DMSO with PEDV was added to Vero cells in 24‐well plates for the first incubation of 1 h. Afterwards, replace the culture supernatant with fresh DMEM and incubate for another 12 h. While PBS was washed, qRT‐PCR was used to measure the mRNA levels of PEDV N and β‐tubulin in the cells.

### 4.5. Virus Attachment Assay

Enoxolone or DMSO‐pretreated Vero cells were incubated for 1 h in a 37°C incubator and then replaced with a mixture of enoxolone or DMSO and PEDV (1 MOI) for incubation at 4°C for 15, 30, and 60 min. Finally, the cells in each well were washed by pre‐cooled PBS, total RNA was collected and reverse transcribed into cDNA. The PEDV viral content was detected by qPCR.

### 4.6. Virus Internalization Assay

Vero cells were infected with PEDV (1 MOI) for 1 h at 4°C. The supernatant was replaced with DMEM containing Enoxolone or DMSO after washed by pre‐cooled PBS, and then incubated for 30 min, 1 h, and 2 h at 37°C. Cells were washed with citrate buffer (pH = 3) to remove the non‐internalized virus, total RNA was collected and reverse transcribed into cDNA. The PEDV viral content was detected by qPCR.

### 4.7. Virus Replication Assay

PEDV (1 MOI) was infected with Vero cells and incubated at 37°C for 1 h in an incubator. PBS was used to wash the cells three times to remove free viruses. The medium was replaced with fresh DMEM containing Enoxolone or DMSO at 4 hpi and incubated at 37°C. Total RNA was collected at 6, 8, and 10 hpi and reverse transcribed into cDNA. The PEDV viral content was detected by qPCR.

### 4.8. Virus Release Assay

Vero cells were infected with PEDV (1 MOI) at 37°C for 1 h. Then, we abandoned virus solution and added fresh DMEM. After 11 h, cells were washed with PBS, and fresh DMEM containing enoxolone or DMSO was added. Cells continued to be cultured at 37°C for 0.5, 1, and 2 h. Total RNA in supernatant and cells was collected simultaneously and reverse transcribed into cDNA. The PEDV viral content was detected by qPCR.

### 4.9. Molecular Docking

Preparation of small molecule ligand and receptor structure files: using I‐TASSER and SWISS‐MODEL two protein modeling online sites for 3D modeling of PEDV proteins with default settings for all parameters. The 3D structure of enoxolone was obtained from PubChem (compound CID: 10114). Autodock or Autodock Vina is used for molecular docking of viral proteins and small molecules. Visualization was performed using Pymol software.

### 4.10. Animal Experiment

The number of 20 3‐day‐old piglets that are maternal antibodies‐free were randomly assigned to four groups (five animals per group); 1 mL PEDV (4 × 10^5^ TCID_50_) was fed to piglets. After 12 hpi, piglets were given 25 and 50 mg/kg body weight of enoxolone. Treatment was consecutive every 6 h until 66 h. After infection, piglets were monitored daily for general health and rectal temperature. All piglets were executed at 72 h. Intestinal and lung tissues were collected for the determination of viral RNA load and histopathological analysis.

### 4.11. Western Blot Assay

Vero cells were washed with PBS and lysed on ice with RIPA for 15 min. Protein samples containing equal amounts were separated by 10% SDS‐PAGE and transferred to PVDF membranes. After transferring, it was closed with 5% skim milk powder (diluted with PBST) for 1 h at room temperature, washed three times with PBST, and then incubated with the following antibodies: anti‐PEDV N protein (1:3000), anti‐β‐tubulin (1:10,000; abmart), anti‐NF‐κB‐p‐p65 (1:10,000, abmart), anti‐NF‐κB‐p65 (1:10,000, abmart), anti‐IκBα (1:10,000, abmart), and anti‐p‐IκBα (1:10,000, abmart). Incubate at room temperature for 2 h or overnight at 4°C. The membranes were then washed with PBST and then incubated with HRP‐conjugated goat anti‐mouse and anti‐rabbit IgG (H + L) secondary antibodies for 1 h at room temperature (1:5000; Beyotime, China). Binding proteins were exposed with an ECL kit (Tanon, China).

### 4.12. RNA Extraction and qRT‐PCR

Total RNA was extracted from cells using the E.Z.N.A. Total RNA Kit (Omega Bio‐Tek, USA) and subsequently reverse transcribed with the HiScript II First Strand cDNA Synthesis Kit (Vazyme, China). Subsequently, qRT‐PCR was carried out using SuperReal PreMix Plus (SYBR Green) (TIANGEN BIOTECH Co., Ltd.) added according to the instructions. RT‐qPCR primers were designed based on the mRNA sequence in NCBI. The primers used in this study are shown in Table 1; three replicates of each reaction were performed, and the data are presented as the mean ± standard deviation (SD).

**Table 1:** Primers used for RT‐qPCR.

Primer	Sequence (5^′^→3^′^)
β‐Actin‐F	CCCAGAGCAAGAGAGGCAT
β‐Actin‐R	GGTAGTCAGTCAGGTCCCG
PEDV‐F	GCAGATTTAGAGCAGCGTTCA
PEDV‐R	TAATCAACCAAACCCACCAC
IL‐6‐F	TGAACTCCTTCTCCACAAGC
IL‐6‐R	GCGGCTACATCTTTGGAATC
IL‐8‐F	CTGGCGGTGGCTCTCTTC
IL‐8‐R	CCTTGGCAAAACTGCACCTT
IL‐1β‐F	GCGGCAACGAGGATGACTT
IL‐1β‐R	TGGCTACAACAACTGACACGG
TNF‐α‐F	GCCACCACGCTCTTCTGTCTG
TNF‐α‐R	AGGGGTCCTTGGGGAACTCTT

### 4.13. Plasmids and Transfection

The PEDV N gene was amplified and cloned into the pCAGGS plasmid with the Flag tag. PCR primers were designed based on the mRNA sequence on NCBI. The transfections were performed according to the protocol of Lipofectamine 3000 (Thermo Fisher Scientific). For the plasmid transfections, Vero cells or HEK‐293T cells were seeded onto 24‐well plates and were then transfected with the PEDV N plasmid. The primers that were used in this study are listed in Table 2.

**Table 2:** Primers used for PCR.

Primer	Sequence (5′→3′)
PEDV‐F	AAAGGTACCATGGCTTCTGTCAGTTTTCAGGATCGT
PEDV‐R	TTTCTCGAGTTACTTATCGTCGTCATCCTTGTAATCATTTCCTGTGTCGAAGAT

### 4.14. Statistical Analysis

GraphPad Prism 9 (GraphPad Software, CA, USA) was employed for statistics. All the experiments in this study were performed independently three times. Western blot was performed, and then grayscale imaging was conducted in ImageJ. Results are shown as the mean ± SD. One‐way or two‐way analysis of variance (ANOVA) was used to compare the means of the groups. Asterisks in the figures show significant differences ( ^∗^ *p* < 0.05;  ^∗∗^ *p* < 0.01;  ^∗∗∗^ *p* < 0.001; ns = not significant).

## Author Contributions

Jie Li participated in the design of the experiments and analyzed the data. Feixiang Zhu and Han Xu was responsible for the in vitro test and part of the piglet challenge experiments and prepared the manuscript. Hongyue Li, Jiang Jin, Muyu Zhu, Junjie Liu for the piglet challenge experiment. Jun Wang, Yingchun Zhou, Zongyi Bo, Haixiao Shen, Junfang Yan performed data statistics and analysis, also assisted in manuscript preparation. Wei Li, Yan Cheng, Xinyue Chang, Xuelan Liu conceived research concepts, Xianrui Zheng, Zongjun Yin, Liang Li and Pei Sun secure research funding, and critically revised the manuscript.

## Funding

This work was supported by the Anhui Higher Institutions Natural Science Foundation, China (Grant 2025AHGXZK20239), the Youth Project of the Natural Science Foundation of Anhui Province, China (Class B) (Grant 2508085Y019), Science and Technology Innovation 2030‐Major Project (Grant 2023ZD0404402‐03), Collaborative Innovation Project of Universities in Anhui Province (Grant GXXT‐2023‐059), the Natural Science Foundation of Anhui Provincial Education Department (Grant 2024AH040083), the National Natural Science Foundation of China (Grants 32202777 and 32573167), the Natural Science Foundation of Anhui Province, China (Grant 2208085MC80), Research Funds of Joint Research Center for Food Nutrition and Health of IHM (Grant 2023SJY01), and the Special Fund for Anhui Agriculture Research System, China (Grants AHCYJSTX‐05‐07 and AHCYJSTX‐05‐21), and the Anhui Agricultural University’s Scientific Research Funding Projects for Introducing and Stabling Talents, China (Grant rc392105).

## Disclosure

All authors discussed the findings and approved the final manuscript.

## Ethics Statement

The experimental protocol for animal testing was strictly followed and approval was obtained from the Ethics Committee of Anhui Agricultural University. All animal experiments were approved by the Animal Care and Ethics Committee of Anhui Agricultural University (Permit Number SYXK 2016‐007) and followed the Guiding Principles for Biomedical Research Involving Animals.

## Conflicts of Interest

The authors declare no conflicts of interest.

## Supporting Information

Additional supporting information can be found online in the Supporting Information section.

## Supporting information


**Supporting Information** Figure S1: A compound named pemetrexed is operated as a positive control targeting PEDV N protein in the molecular docking analyze. Using Pymol to visualize the docking result between pemetrexed and PEDV N protein.

## Data Availability

The datasets employed in this study can be obtained upon a reasonable request directed to the corresponding author.
